# Crystal structures of three hydrogen-bonded 1:2 compounds of chloranilic acid with 2-pyridone, 3-hy­droxy­pyridine and 4-hyroxypyridine

**DOI:** 10.1107/S2056989017013536

**Published:** 2017-09-29

**Authors:** Kazuma Gotoh, Hiroyuki Ishida

**Affiliations:** a Department of Chemistry, Faculty of Science, Okayama University, Okayama 700-8530, Japan

**Keywords:** crystal structure, chloranilic acid, 2-pyridone, 3-hy­droxy­pyridine, 4-hyroxypyridine, hydrogen bond

## Abstract

The crystal structures of the 1:2 compounds of chloranilic acid (systematic name: 2,5-di­chloro-3,6-dihy­droxy-1,4-benzo­quinone) with 2-pyridone, 3-hy­droxy­pyridine and 4-hyroxypyridine, namely, bis­(2-pyridone) chloranilic acid, 2C_5_H_5_NO·C_6_H_2_Cl_2_O_4_, (I), bis­(3-hy­droxy­pyridinium) chloranilate, 2C_5_H_6_NO^+^·C_6_Cl_2_O_4_^2−^, (II), and bis­(4-hy­droxy­pyridinium) chloranilate, 2C_5_H_6_NO^+^·C_6_Cl_2_O_4_^2−^, (III), have been determined at 120 K. In the crystal of (I), the base mol­ecule is in the lactam form and no acid–base inter­action involving H-atom transfer is observed. The acid mol­ecule lies on an inversion centre and the asymmetric unit consists of one half-mol­ecule of chloranilic acid and one 2-pyridone mol­ecule, which are linked *via* a short O—H⋯O hydrogen bond. 2-Pyridone mol­ecules form a head-to-head dimer *via* a pair of N—H⋯O hydrogen bonds, resulting in a tape structure along [201]. In the crystals of (II) and (III), acid–base inter­actions involving H-atom transfer are observed and the divalent cations lie on an inversion centre. The asymmetric unit of (II) consists of one half of a chloranilate anion and one 3-hy­droxy­pyridinium cation, while that of (III) comprises two independent halves of anions and two 4-hy­droxy­pyridinium cations. The primary inter­molecular inter­action in (II) is a bifurcated O—H⋯(O,O) hydrogen bond between the cation and the anion. The hydrogen-bonded units are further linked *via* N—H⋯O hydrogen bonds, forming a layer parallel to the *bc* plane. In (III), one anion is surrounded by four cations *via* O—H⋯O and C—H⋯O hydrogen bonds, while the other is surrounded by four cations *via* N—H⋯O and C—H⋯Cl hydrogen bonds. These inter­actions link the cations and the anions into a layer parallel to (301).

## Abstract 2

Crystal structures of hydrogen-bonded 1:2 compounds of chloranilic acid with 2-pyridone, 3-hy­droxy­pyridine and 4-hyroxypyridine have been determined at 120 K. In each crystal structure, the acid and base mol­ecules are linked by short O—H⋯O and N—H⋯O hydrogen bonds.

## Chemical context   

Chloranilic acid, a dibasic acid with hydrogen-bond donor and acceptor groups, appears particularly attractive as a template for generating tightly bound self-assemblies with various pyridine derivatives, as well as a model compound for investigating hydrogen transfer motions in O—H⋯N and N—H⋯O hydrogen-bond systems (Zaman *et al.*, 2004 ▸; Seliger *et al.*, 2009 ▸; Asaji *et al.* 2010 ▸). In the present study, we have prepared three 1:2 compounds of chloranilic acid with 2-pyridone, 3-hy­droxy­pyridine and 4-hy­droxy­pyridine in order to extend our study of *D*—H⋯*A* hydrogen bonding (*D* = N, O or C; *A* = N, O or Cl) in chloranilic acid–substituted-pyridine systems (Gotoh *et al.*, 2009*a* ▸,*b* ▸, 2010 ▸). The crystal structure of the 1:1 compound of chloranilic acid with 3-hy­droxy­pyridine, namely, 3-hy­droxy­pyridinium hydrogen chloranilate monohydrate, has been reported (Gotoh & Ishida, 2009 ▸).
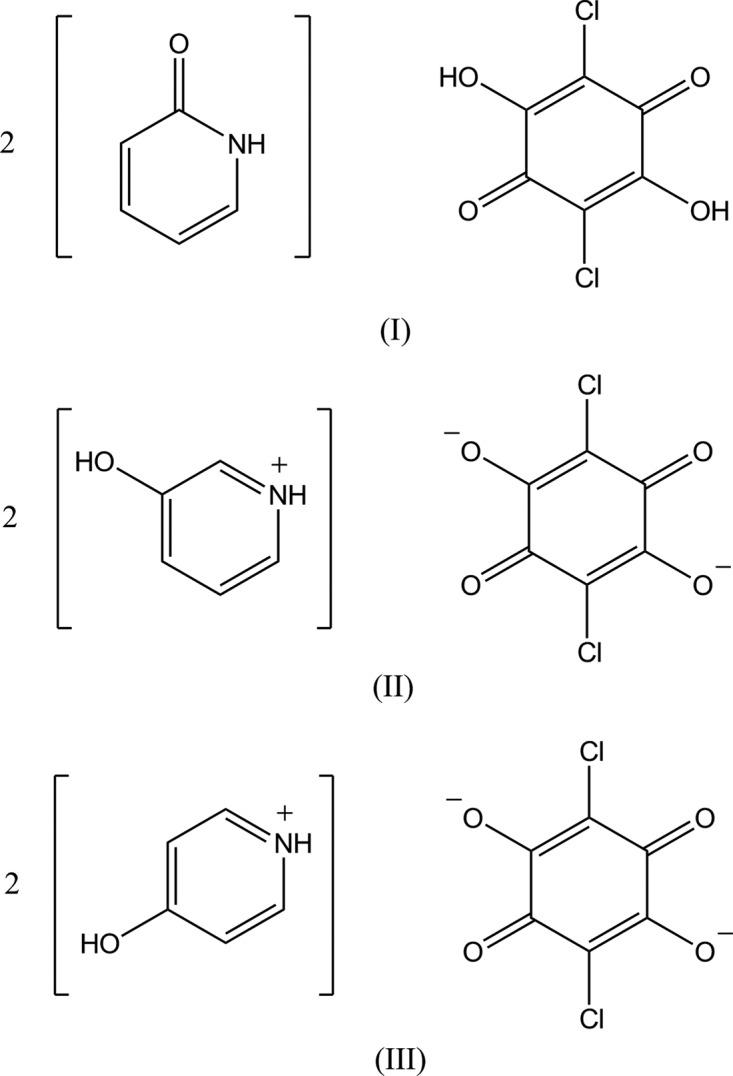


## Structural commentary   

**Figure 1:**
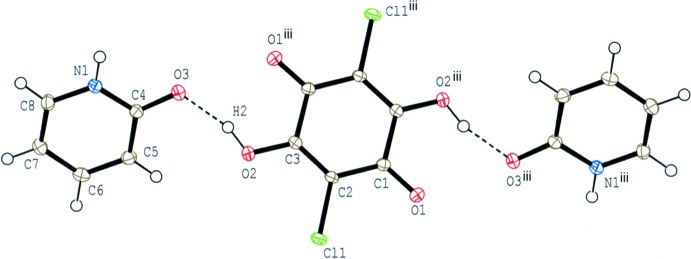
The mol­ecular structure of compound (I), showing the atom-numbering scheme. Displacement ellipsoids are drawn at the 50% probability level and H atoms are shown as small spheres of arbitrary radii. O—H⋯O hydrogen bonds are shown as dashed lines. [Symmetry code: (iii) −*x* + 1, −*y* + 1, −*z* + 1.]

In compound (I), the base mol­ecule is in the lactam form and no acid-base inter­action involving H-atom transfer is observed (Fig. 1 ▸). The acid mol­ecule lies on an inversion centre and the asymmetric unit consists of one-half acid mol­ecule and one base mol­ecule, which are linked *via* a short O—H⋯O hydrogen bond (O2—H2⋯O3; Table 1 ▸). The dihedral angle between the acid ring and the base ring is 37.82 (5)°.

**Figure 2:**
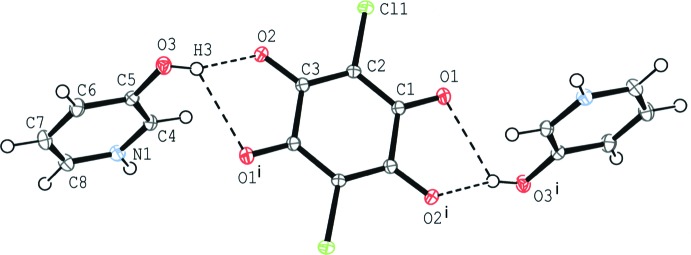
The mol­ecular structure of compound (II), showing the atom-numbering scheme. Displacement ellipsoids are drawn at the 50% probability level and H atoms are shown as small spheres of arbitrary radii. O—H⋯O hydrogen bonds are shown as dashed lines. [Symmetry code: (i) −*x* + 2, −*y* + 1, −*z* + 1.]

In compound (II), an acid–base inter­action involving H-atom transfer is observed. The chloranilate anion is located on an inversion centre and the asymmetric unit contains one-half anion mol­ecule and one cation mol­ecule. The primary inter­molecular inter­action between the cation and the anion is a bifurcated O—H⋯(O,O) hydrogen bond (O3—H3⋯O2 and O3—H3⋯O1^i^; symmetry code as in Table 2 ▸) to afford a centrosymmetric 1:2 aggregate of the anion and the cation (Fig. 2 ▸). The dihedral angle between the acid ring and the base ring is 72.69 (5)°.

**Figure 3:**
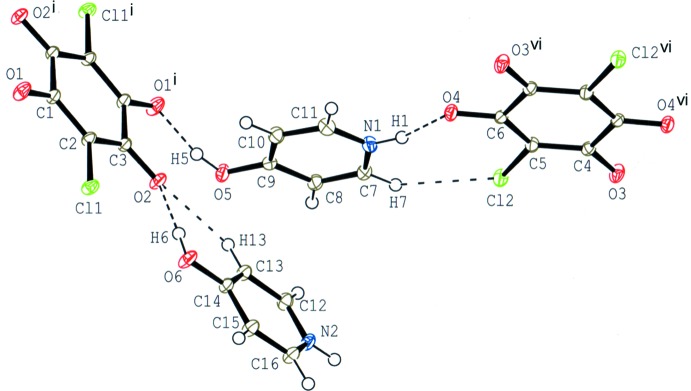
The mol­ecular structure of compound (III), showing the atom-numbering scheme. Displacement ellipsoids are drawn at the 50% probability level and H atoms are shown as small spheres of arbitrary radii. O—H⋯O, N—H⋯O, C—H⋯Cl and C—H⋯O hydrogen bonds are shown as dashed lines. [Symmetry codes: (i) −*x*, −*y*, −*z*; (vi) −*x* + 3, −*y*, −*z* + 1.]

**Figure 4:**
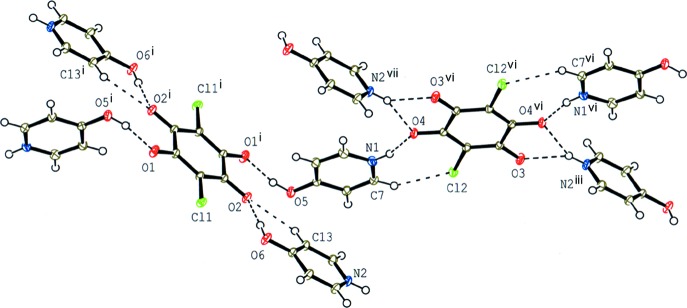
A partial packing diagram for compound (III) around two independent chloranilate anions. O—H⋯O, N—H⋯O, C—H⋯Cl and C—H⋯O hydrogen bonds are shown as dashed lines. [Symmetry codes: (i) −*x*, −*y*, −*z*; (iii) −*x* + 3, −*y* + 1, −*z* + 1; (vi) −*x* + 3, −*y*, −*z* + 1; (vii) *x*, *y* − 1, *z*.]

The compound (III) crystallizes with two independent halves of chloranilate anions and two 4-hy­droxy­pyridinium cations in the asymmetric unit (Fig. 3 ▸). Although both anions lie on an inversion centre, the hydrogen-bonding schemes around the anions are quite different (Fig. 4 ▸); one anion is surrounded by four cations *via* O—H⋯O and C—H⋯O hydrogen bonds (O5—H5⋯O1^i^, O6—H6⋯O2 and C13—H13⋯O2; symmetry code as in Table 3 ▸), while the other is surrounded by four cation *via* N—H⋯O and C—H⋯Cl hydrogen bonds (N1—H1⋯O4, N2—H2⋯O4^ii^, N2—H2⋯O3^iii^ and C7—H7⋯Cl2; Table 3 ▸).

## Supra­molecular features   

**Figure 5:**
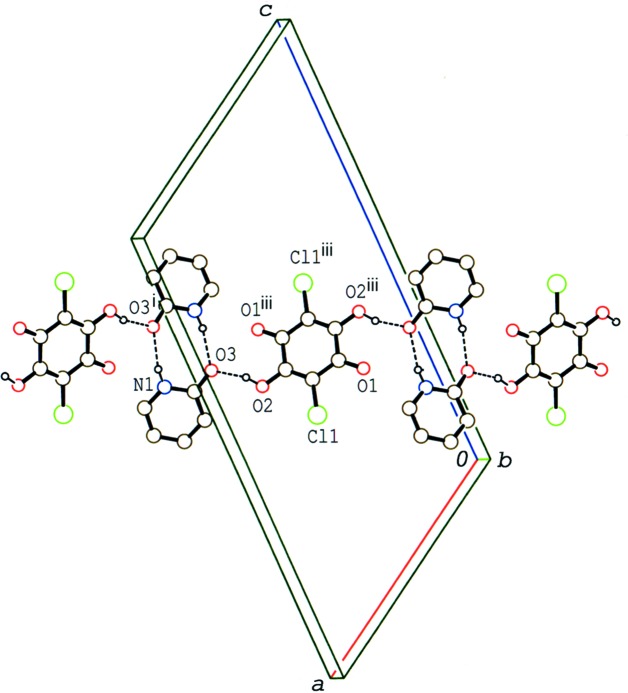
A packing diagram for compound (I), showing the tape structure formed by O—H⋯O and N—H⋯O hydrogen bonds (dashed lines). H atoms not involved in the inter­actions have been omitted. [Symmetry codes: (i) −*x* + 2, *y*, −*z* + 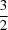; (iii) −*x* + 1, −*y* + 1, −*z* + 1.]

In the crystal of compound (I), two adjacent 2-pyridone mol­ecules, which are related by a twofold rotation axis, form a head-to-head dimer *via* a pair of N—H⋯O hydrogen bonds (N1—H1⋯O3^i^; symmetry code as in Table 1 ▸), as observed in various cocrystals of 2-pyridone (Odani & Matsumoto, 2002 ▸). The acid and base mol­ecules form an undulating tape structure running along [201] through the above-mentioned O—H⋯O and N—H⋯O hydrogen bonds (Fig. 5 ▸). The tapes are stacked along the *b* axis into a layer structure through a π–π inter­action between the pyridine rings [centroid-to-centroid distance = 3.7005 (6) Å and inter­planar spacing = 3.4239 (4) Å] and a short C⋯C contact [C2⋯C3^iv^ = 3.3056 (13) Å; symmetry code: (iv) *x*, *y* + 1, *z*]. A weak C—H⋯Cl inter­action formed between the acid and base mol­ecules (C7—H7⋯Cl1^ii^; Table 1 ▸) links the layers. The O—H⋯O hydrogen bond between the acid and base mol­ecules is short [O2⋯O3 = 2.4989 (11) Å], suggesting possible disorder of the H atom in the hydrogen bond, but no distinct evidence of the disorder was observed in the difference Fourier map, nor from the mol­ecular geometry.

**Figure 6:**
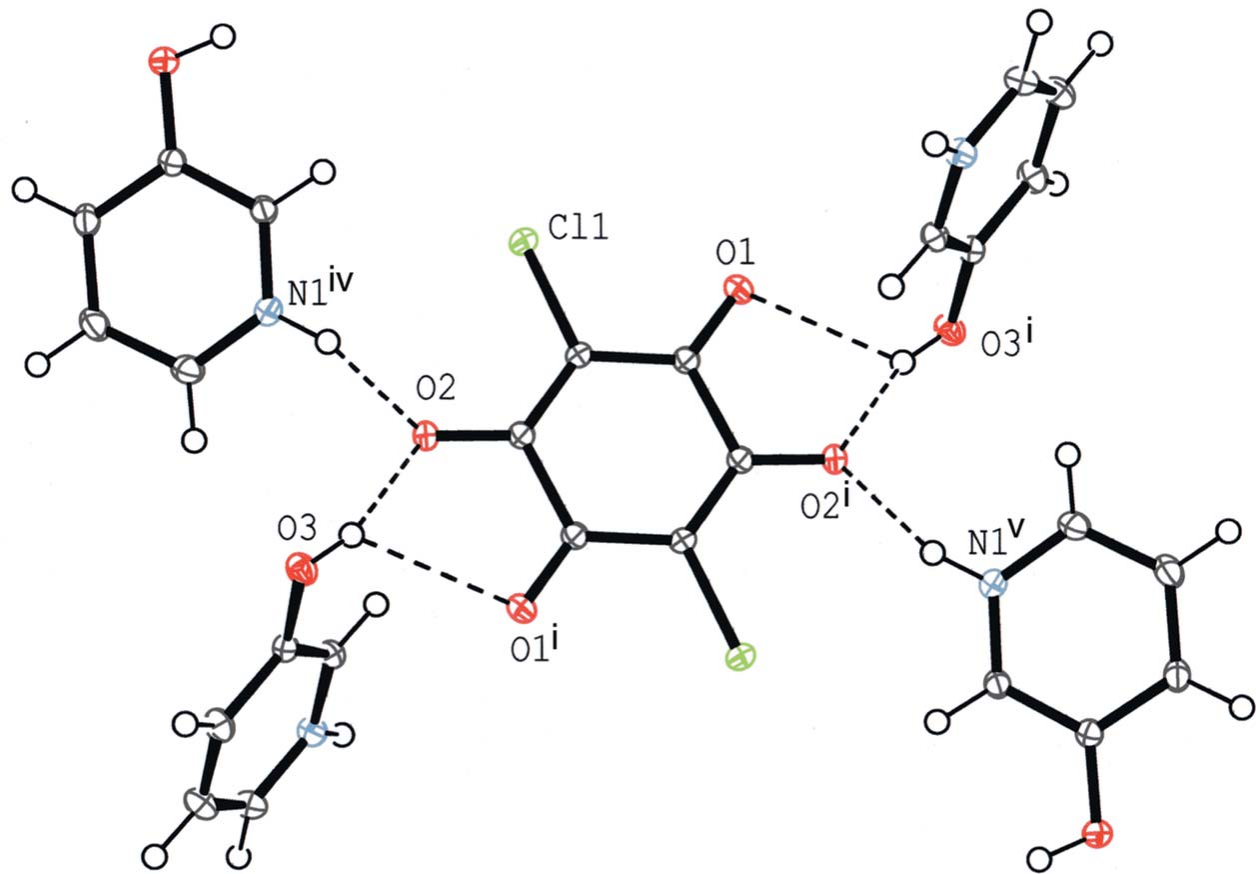
A partial packing diagram for compound (II) around the chloranilate anion. O—H⋯O and N—H⋯O hydrogen bonds are shown as dashed lines. [Symmetry codes: (i) −*x* + 2, −*y* + 1, −*z* + 1; (iv) *x*, −*y* + 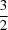, *z* + 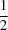; (v) −*x* + 2, *y* − 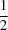, −*z* + 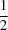.]

**Figure 7:**
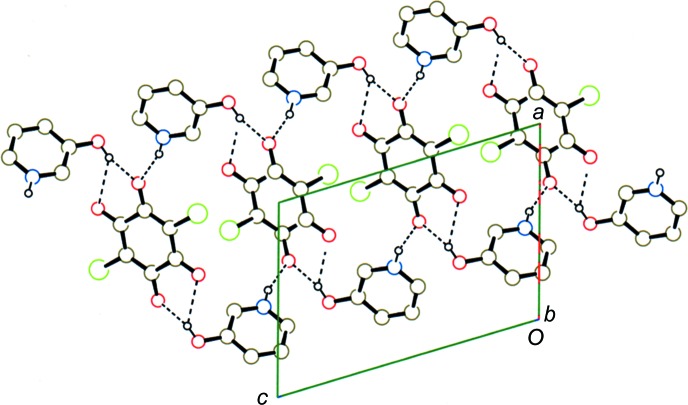
A packing diagram for compound (II), viewed along the *b* axis, showing the layer structure formed by O—H⋯O and N—H⋯O hydrogen bonds (dashed lines). H atoms not involved in the inter­actions have been omitted.

In the crystal of (II), the cation–anion units are further connected by N—H⋯O (N1—H1⋯O2^ii^; symmetry code as in Table 2 ▸ and Fig. 6 ▸), forming a layer expanding parallel to the *bc* plane (Fig. 7 ▸). Adjacent layers are connected to each other with a C—H⋯O hydrogen bond (C8—H8⋯O1^iii^; Table 2 ▸) and a short O⋯N contact [O3⋯N1^vi^ = 3.0430 (12) Å; symmetry code: (vi) −*x* + 1, *y* − 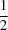, −*z* + 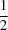].

**Figure 8:**
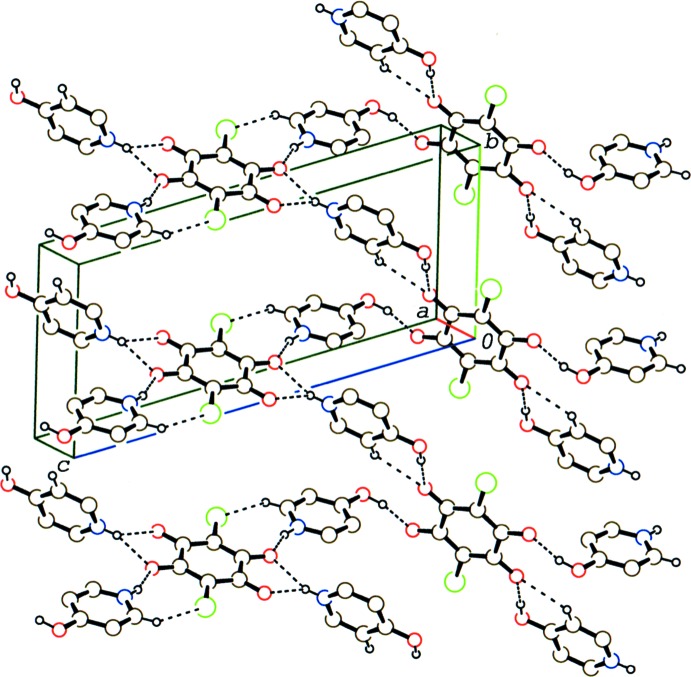
A packing diagram for compound (III), showing the hydrogen-bonded network in the layer. O—H⋯O, N—H⋯O, C—H⋯Cl and C—H⋯O hydrogen bonds are shown as dashed lines. H atoms not involved in the hydrogen bonds have been omitted.

In the crystal of (III), the above-mentioned O—H⋯O, N—H⋯O, C—H⋯O and C—H⋯Cl hydrogen bonds link the cations and anions into a layer parallel to (301) (Fig. 8 ▸). Adjacent layers are further linked *via* weak C—H⋯O and C—H⋯Cl inter­actions (C12—H12⋯O3^iv^ and C16—H16⋯Cl1^v^; symmetry codes as given in Table 3 ▸).

## Database survey   

A search of the Cambridge Structural Database (Version 5.38, last update May 2017; Groom *et al.*, 2016 ▸) for organic crystals of chloranilic acid with substituted pyridines (except for di-, tri- and tetra­pyridine derivatives) gave 32 hits. Of these, crystal structures of 16 compounds of chloranilic acid with methyl-substituted pyridines (Adam *et al.*, 2010 ▸; Łuczyńska *et al.*, 2016 ▸; Molčanov & Kojić-Prodić, 2010 ▸, and references therein), three compounds of carbamoyl-substituted pyridines (Gotoh *et al.*, 2009*a* ▸), three compounds of carb­oxy-substituted pyridines (Gotoh *et al.*, 2009*b* ▸, and references therein) and three compounds of cyano-substituted pyridines (Gotoh & Ishida, 2012 ▸, and references therein) were reported.

## Synthesis and crystallization   

Single crystals of compound (I) were obtained by slow evaporation from an ethanol solution (120 ml) of chloranilic acid (350 mg) with 2-hy­droxy­pyridine (340 mg) at room temperature. Crystals of compound (II) were obtained by slow evaporation from a methanol solution (400 ml) of chloranilic acid (170 mg) with 3-hy­droxy­pyridine (160 mg) at room temperature. Crystals of compound (III) were obtained by slow diffusion of a methanol solution (20 ml) of 4-hy­droxy­pyridine (160 mg) into an aceto­nitrile solution (200 ml) of chloranilic acid (170 mg) at room temperature.

## Refinement   

Crystal data, data collection and structure refinement details are summarized in Table 4 ▸. All H atoms in compounds (I)–(III) were found in difference Fourier maps. The positions of O- and N-bound H atoms were refined freely, with *U*_iso_(H) = 1.5*U*_eq_(O or N). C-bound H atoms were positioned geometrically (C—H = 0.95 Å) and were treated as riding with *U*_iso_(H) = 1.2*U*_eq_(C).

## Supplementary Material

Crystal structure: contains datablock(s) I, II, III, global. DOI: 10.1107/S2056989017013536/lh5855sup1.cif

Structure factors: contains datablock(s) I. DOI: 10.1107/S2056989017013536/lh5855Isup2.hkl

Structure factors: contains datablock(s) II. DOI: 10.1107/S2056989017013536/lh5855IIsup3.hkl

Structure factors: contains datablock(s) III. DOI: 10.1107/S2056989017013536/lh5855IIIsup4.hkl

CCDC references: 1575722, 1575721, 1575720

Additional supporting information:  crystallographic information; 3D view; checkCIF report

## Figures and Tables

**Table 1:** Hydrogen-bond geometry (Å, °) for (I)

*D*—H⋯*A*	*D*—H	H⋯*A*	*D*⋯*A*	*D*—H⋯*A*
O2—H2⋯O3	0.901 (15)	1.627 (16)	2.4989 (11)	161.9 (19)
N1—H1⋯O3^i^	0.893 (16)	1.996 (17)	2.8743 (12)	167.6 (16)
C7—H7⋯Cl1^ii^	0.95	2.79	3.5122 (13)	134

Symmetry codes: (i) 
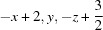
; (ii) 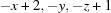.

**Table 2:** Hydrogen-bond geometry (Å, °) for (II)

*D*—H⋯*A*	*D*—H	H⋯*A*	*D*⋯*A*	*D*—H⋯*A*
O3—H3⋯O2	0.852 (17)	1.803 (17)	2.6277 (12)	162.5 (16)
O3—H3⋯O1^i^	0.852 (17)	2.438 (17)	2.9738 (12)	121.6 (14)
N1—H1⋯O2^ii^	0.889 (17)	1.807 (17)	2.6684 (12)	162.6 (16)
C8—H8⋯O1^iii^	0.95	2.45	3.1481 (13)	130

Symmetry codes: (i) 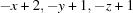; (ii) 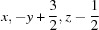; (iii) 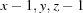.

**Table 3:** Hydrogen-bond geometry (Å, °) for (III)

*D*—H⋯*A*	*D*—H	H⋯*A*	*D*⋯*A*	*D*—H⋯*A*
O5—H5⋯O1^i^	0.905 (17)	1.640 (17)	2.5208 (13)	163.2 (17)
O6—H6⋯O2	0.852 (17)	1.800 (17)	2.6510 (13)	177.3 (18)
N1—H1⋯O4	0.919 (17)	1.810 (17)	2.7000 (13)	162.3 (16)
N2—H2⋯O4^ii^	0.876 (18)	2.156 (17)	2.9603 (14)	152.3 (15)
N2—H2⋯O3^iii^	0.876 (18)	2.176 (17)	2.8384 (14)	132.1 (14)
C7—H7⋯Cl2	0.95	2.81	3.4540 (12)	126
C12—H12⋯O3^iv^	0.95	2.32	3.1541 (14)	146
C13—H13⋯O2	0.95	2.49	3.1685 (14)	128
C16—H16⋯Cl1^v^	0.95	2.77	3.4427 (12)	128

Symmetry codes: (i) 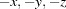; (ii) 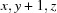; (iii) 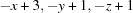; (iv) 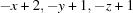; (v) 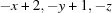.

**Table 4:** Experimental details

	(I)	(II)	(III)
Crystal data			
Chemical formula	2C_5_H_5_NO·C_6_H_2_Cl_2_O_4_	2C_5_H_6_NO^+^·C_6_Cl_2_O_4_^2−^	2C_5_H_6_NO^+^·C_6_Cl_2_O_4_^2−^
*M*_r_	399.19	399.19	399.19
Crystal system, space group	Monoclinic, *P*2/*c*	Monoclinic, *P*2_1_/*c*	Triclinic, *P* 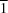
Temperature (K)	120	120	120
*a*, *b*, *c* (Å)	11.9402 (7), 3.7005 (2), 21.7919 (13)	8.3659 (6), 8.5492 (6), 11.7087 (8)	5.49136 (13), 8.2195 (4), 18.1382 (9)
α, β, γ (°)	90, 121.278 (2), 90	90, 106.968 (3), 90	102.177 (3), 93.952 (3), 95.316 (4)
*V* (Å^3^)	822.92 (9)	800.98 (9)	793.52 (6)
*Z*	2	2	2
Radiation type	Mo *K*α	Mo *K*α	Mo *K*α
μ (mm^−1^)	0.43	0.45	0.45
Crystal size (mm)	0.39 × 0.36 × 0.21	0.21 × 0.20 × 0.12	0.35 × 0.25 × 0.12

Data collection			
Diffractometer	Rigaku R-AXIS RAPIDII	Rigaku R-AXIS RAPIDII	Rigaku R-AXIS RAPIDII
Absorption correction	Multi-scan (*ABSCOR*; Higashi, 1995 ▸)	Numerical (*NUMABS*; Higashi, 1999 ▸)	Numerical (*NUMABS*; Higashi, 1999 ▸)
*T*_min_, *T*_max_	0.804, 0.913	0.903, 0.948	0.890, 0.948
No. of measured, independent and observed [*I* > 2σ(*I*)] reflections	22767, 2401, 2316	15315, 2329, 2166	12373, 4597, 4124
*R*_int_	0.013	0.017	0.036
(sin θ/λ)_max_ (Å^−1^)	0.703	0.703	0.703

Refinement			
*R*[*F* ^2^ > 2σ(*F* ^2^)], *wR*(*F* ^2^), *S*	0.027, 0.076, 1.06	0.028, 0.075, 1.07	0.030, 0.080, 1.07
No. of reflections	2401	2329	4597
No. of parameters	124	124	247
H-atom treatment	H atoms treated by a mixture of independent and constrained refinement	H atoms treated by a mixture of independent and constrained refinement	H atoms treated by a mixture of independent and constrained refinement
Δρ_max_, Δρ_min_ (e Å^−3^)	0.49, −0.24	0.52, −0.20	0.75, −0.34

Computer programs: *RAPID-AUTO* (Rigaku, 2006 ▸), *SHELXS97* (Sheldrick, 2008 ▸), *SHELXL2016* (Sheldrick, 2015 ▸), *ORTEP-3 for Windows* (Farrugia, 2012 ▸), *CrystalStructure* (Rigaku, 2010 ▸) and *PLATON* (Spek, 2009 ▸).

## supplementary crystallographic information

### Bis[pyridin-2(1H)-one] 2,5-dichloro-3,6-dihydroxy-1,4-bebzoquinone (I) Crystal data

**Table:**

2C_5_H_5_NO·C_6_H_2_Cl_2_O_4_	*F*(000) = 408.00
*M**_r_* = 399.19	*D*_x_ = 1.611 Mg m^−^^3^
Monoclinic, *P*2/*c*	Mo *K*α radiation, λ = 0.71075 Å
*a* = 11.9402 (7) Å	Cell parameters from 20111 reflections
*b* = 3.7005 (2) Å	θ = 3.3–30.1°
*c* = 21.7919 (13) Å	µ = 0.43 mm^−^^1^
β = 121.278 (2)°	*T* = 120 K
*V* = 822.92 (9) Å^3^	Block, brown
*Z* = 2	0.39 × 0.36 × 0.21 mm

### Bis[pyridin-2(1H)-one] 2,5-dichloro-3,6-dihydroxy-1,4-bebzoquinone (I) Data collection

**Table:**

Rigaku R-AXIS RAPIDII diffractometer	2316 reflections with *I* > 2σ(*I*)
Detector resolution: 10.000 pixels mm^-1^	*R*_int_ = 0.013
ω scans	θ_max_ = 30.0°, θ_min_ = 3.4°
Absorption correction: multi-scan (ABSCOR; Higashi, 1995)	*h* = −16→16
*T*_min_ = 0.804, *T*_max_ = 0.913	*k* = −4→5
22767 measured reflections	*l* = −30→30
2401 independent reflections	

### Bis[pyridin-2(1H)-one] 2,5-dichloro-3,6-dihydroxy-1,4-bebzoquinone (I) Refinement

**Table:**

Refinement on *F*^2^	Primary atom site location: structure-invariant direct methods
Least-squares matrix: full	Secondary atom site location: difference Fourier map
*R*[*F*^2^ > 2σ(*F*^2^)] = 0.027	Hydrogen site location: mixed
*wR*(*F*^2^) = 0.076	H atoms treated by a mixture of independent and constrained refinement
*S* = 1.06	*w* = 1/[σ^2^(*F*_o_^2^) + (0.0443*P*)^2^ + 0.3557*P*] where *P* = (*F*_o_^2^ + 2*F*_c_^2^)/3
2401 reflections	(Δ/σ)_max_ = 0.001
124 parameters	Δρ_max_ = 0.49 e Å^−^^3^
0 restraints	Δρ_min_ = −0.24 e Å^−^^3^

### Bis[pyridin-2(1H)-one] 2,5-dichloro-3,6-dihydroxy-1,4-bebzoquinone (I) Special details

**Table:**

Geometry. All esds (except the esd in the dihedral angle between two l.s. planes) are estimated using the full covariance matrix. The cell esds are taken into account individually in the estimation of esds in distances, angles and torsion angles; correlations between esds in cell parameters are only used when they are defined by crystal symmetry. An approximate (isotropic) treatment of cell esds is used for estimating esds involving l.s. planes.

### Bis[pyridin-2(1H)-one] 2,5-dichloro-3,6-dihydroxy-1,4-bebzoquinone (I) Fractional atomic coordinates and isotropic or equivalent isotropic displacement parameters (Å^2^)

**Table:**

	*x*	*y*	*z*	*U*_iso_*/*U*_eq_	
Cl1	0.60869 (2)	0.78653 (6)	0.40403 (2)	0.01704 (8)	
O1	0.33921 (7)	0.8229 (2)	0.37552 (4)	0.02149 (16)	
O2	0.75999 (6)	0.4050 (2)	0.54453 (4)	0.01794 (15)	
H2	0.8039 (15)	0.289 (4)	0.5870 (8)	0.027*	
O3	0.92219 (7)	0.1199 (2)	0.66164 (4)	0.02094 (16)	
N1	1.13199 (8)	−0.0736 (2)	0.72858 (4)	0.01604 (16)	
H1	1.1281 (13)	−0.016 (4)	0.7672 (8)	0.024*	
C1	0.41591 (9)	0.6765 (2)	0.43248 (5)	0.01361 (17)	
C2	0.55361 (8)	0.6274 (3)	0.45806 (5)	0.01311 (16)	
C3	0.63639 (8)	0.4587 (2)	0.52136 (5)	0.01342 (17)	
C4	1.02174 (9)	−0.0153 (3)	0.66317 (5)	0.01563 (17)	
C5	1.02800 (9)	−0.1116 (3)	0.60168 (5)	0.01779 (18)	
H5	0.953363	−0.080221	0.554948	0.021*	
C6	1.14171 (10)	−0.2497 (3)	0.60990 (5)	0.01837 (19)	
H6	1.145376	−0.312374	0.568705	0.022*	
C7	1.25330 (10)	−0.2995 (3)	0.67902 (5)	0.01870 (19)	
H7	1.332337	−0.392760	0.684778	0.022*	
C8	1.24515 (9)	−0.2109 (3)	0.73732 (5)	0.01775 (18)	
H8	1.318846	−0.245048	0.784277	0.021*	

### Bis[pyridin-2(1H)-one] 2,5-dichloro-3,6-dihydroxy-1,4-bebzoquinone (I) Atomic displacement parameters (Å^2^)

**Table:**

	*U*^11^	*U*^22^	*U*^33^	*U*^12^	*U*^13^	*U*^23^
Cl1	0.02123 (12)	0.01778 (12)	0.01883 (12)	0.00121 (8)	0.01512 (10)	0.00259 (7)
O1	0.0172 (3)	0.0304 (4)	0.0166 (3)	0.0046 (3)	0.0086 (3)	0.0080 (3)
O2	0.0124 (3)	0.0244 (4)	0.0172 (3)	0.0022 (3)	0.0079 (2)	0.0040 (3)
O3	0.0149 (3)	0.0307 (4)	0.0184 (3)	0.0048 (3)	0.0095 (3)	0.0034 (3)
N1	0.0158 (3)	0.0189 (4)	0.0145 (3)	0.0021 (3)	0.0086 (3)	0.0000 (3)
C1	0.0150 (4)	0.0138 (4)	0.0134 (4)	0.0000 (3)	0.0083 (3)	−0.0005 (3)
C2	0.0150 (4)	0.0139 (4)	0.0139 (4)	0.0000 (3)	0.0098 (3)	0.0002 (3)
C3	0.0139 (4)	0.0137 (4)	0.0143 (4)	−0.0005 (3)	0.0084 (3)	−0.0010 (3)
C4	0.0142 (4)	0.0170 (4)	0.0162 (4)	−0.0005 (3)	0.0083 (3)	0.0012 (3)
C5	0.0178 (4)	0.0207 (4)	0.0146 (4)	−0.0011 (3)	0.0082 (3)	0.0004 (3)
C6	0.0221 (4)	0.0189 (4)	0.0179 (4)	−0.0015 (3)	0.0131 (4)	−0.0018 (3)
C7	0.0184 (4)	0.0190 (4)	0.0211 (4)	0.0023 (3)	0.0119 (4)	−0.0009 (3)
C8	0.0157 (4)	0.0191 (4)	0.0174 (4)	0.0033 (3)	0.0078 (3)	0.0002 (3)

### Bis[pyridin-2(1H)-one] 2,5-dichloro-3,6-dihydroxy-1,4-bebzoquinone (I) Geometric parameters (Å, º)

**Table:**

Cl1—C2	1.7233 (9)	C2—C3	1.3614 (12)
O1—C1	1.2217 (11)	C4—C5	1.4256 (13)
O2—C3	1.3033 (10)	C5—C6	1.3726 (14)
O2—H2	0.901 (16)	C5—H5	0.9500
O3—C4	1.2739 (11)	C6—C7	1.4120 (14)
N1—C8	1.3611 (12)	C6—H6	0.9500
N1—C4	1.3648 (11)	C7—C8	1.3636 (14)
N1—H1	0.892 (15)	C7—H7	0.9500
C1—C2	1.4475 (12)	C8—H8	0.9500
C1—C3^i^	1.5182 (12)		
			
C3—O2—H2	114.1 (10)	O3—C4—C5	125.24 (8)
C8—N1—C4	123.66 (8)	N1—C4—C5	116.69 (8)
C8—N1—H1	119.5 (9)	C6—C5—C4	120.10 (9)
C4—N1—H1	116.9 (9)	C6—C5—H5	120.0
O1—C1—C2	123.32 (8)	C4—C5—H5	120.0
O1—C1—C3^i^	118.05 (8)	C5—C6—C7	120.62 (9)
C2—C1—C3^i^	118.63 (7)	C5—C6—H6	119.7
C3—C2—C1	121.99 (8)	C7—C6—H6	119.7
C3—C2—Cl1	121.00 (7)	C8—C7—C6	118.57 (9)
C1—C2—Cl1	117.01 (7)	C8—C7—H7	120.7
O2—C3—C2	122.88 (8)	C6—C7—H7	120.7
O2—C3—C1^i^	117.75 (8)	N1—C8—C7	120.35 (9)
C2—C3—C1^i^	119.37 (8)	N1—C8—H8	119.8
O3—C4—N1	118.06 (8)	C7—C8—H8	119.8
			
O1—C1—C2—C3	−179.08 (9)	C8—N1—C4—O3	−178.90 (9)
C3^i^—C1—C2—C3	1.18 (15)	C8—N1—C4—C5	1.04 (15)
O1—C1—C2—Cl1	−0.05 (13)	O3—C4—C5—C6	178.84 (10)
C3^i^—C1—C2—Cl1	−179.79 (6)	N1—C4—C5—C6	−1.09 (15)
C1—C2—C3—O2	178.46 (9)	C4—C5—C6—C7	0.28 (16)
Cl1—C2—C3—O2	−0.53 (14)	C5—C6—C7—C8	0.64 (16)
C1—C2—C3—C1^i^	−1.19 (15)	C4—N1—C8—C7	−0.13 (16)
Cl1—C2—C3—C1^i^	179.82 (7)	C6—C7—C8—N1	−0.73 (15)

Symmetry code: (i) −*x*+1, −*y*+1, −*z*+1.

### Bis[pyridin-2(1H)-one] 2,5-dichloro-3,6-dihydroxy-1,4-bebzoquinone (I) Hydrogen-bond geometry (Å, º)

**Table:**

*D*—H···*A*	*D*—H	H···*A*	*D*···*A*	*D*—H···*A*
O2—H2···O3	0.901 (15)	1.627 (16)	2.4989 (11)	161.9 (19)
N1—H1···O3^ii^	0.893 (16)	1.996 (17)	2.8743 (12)	167.6 (16)
C7—H7···Cl1^iii^	0.95	2.79	3.5122 (13)	134

Symmetry codes: (ii) −*x*+2, *y*, −*z*+3/2; (iii) −*x*+2, −*y*, −*z*+1.

### Bis(3-hyroxypyridinium) 2,5-dichloro-3,6-dioxocyclohexa-1,4-diene-1,4-diolate (II) Crystal data

**Table:**

2C_5_H_6_NO^+^·C_6_Cl_2_O_4_^2^^−^	*F*(000) = 408.00
*M**_r_* = 399.19	*D*_x_ = 1.655 Mg m^−^^3^
Monoclinic, *P*2_1_/*c*	Mo *K*α radiation, λ = 0.71075 Å
*a* = 8.3659 (6) Å	Cell parameters from 13386 reflections
*b* = 8.5492 (6) Å	θ = 3.0–30.0°
*c* = 11.7087 (8) Å	µ = 0.45 mm^−^^1^
β = 106.968 (3)°	*T* = 120 K
*V* = 800.98 (9) Å^3^	Block, brown
*Z* = 2	0.21 × 0.20 × 0.12 mm

### Bis(3-hyroxypyridinium) 2,5-dichloro-3,6-dioxocyclohexa-1,4-diene-1,4-diolate (II) Data collection

**Table:**

Rigaku R-AXIS RAPIDII diffractometer	2166 reflections with *I* > 2σ(*I*)
Detector resolution: 10.000 pixels mm^-1^	*R*_int_ = 0.017
ω scans	θ_max_ = 30.0°, θ_min_ = 3.0°
Absorption correction: numerical (*NUMABS*; Higashi, 1999)	*h* = −11→11
*T*_min_ = 0.903, *T*_max_ = 0.948	*k* = −12→12
15315 measured reflections	*l* = −15→16
2329 independent reflections	

### Bis(3-hyroxypyridinium) 2,5-dichloro-3,6-dioxocyclohexa-1,4-diene-1,4-diolate (II) Refinement

**Table:**

Refinement on *F*^2^	Primary atom site location: structure-invariant direct methods
Least-squares matrix: full	Secondary atom site location: difference Fourier map
*R*[*F*^2^ > 2σ(*F*^2^)] = 0.028	Hydrogen site location: mixed
*wR*(*F*^2^) = 0.075	H atoms treated by a mixture of independent and constrained refinement
*S* = 1.07	*w* = 1/[σ^2^(*F*_o_^2^) + (0.0411*P*)^2^ + 0.357*P*] where *P* = (*F*_o_^2^ + 2*F*_c_^2^)/3
2329 reflections	(Δ/σ)_max_ = 0.001
124 parameters	Δρ_max_ = 0.52 e Å^−^^3^
0 restraints	Δρ_min_ = −0.20 e Å^−^^3^

### Bis(3-hyroxypyridinium) 2,5-dichloro-3,6-dioxocyclohexa-1,4-diene-1,4-diolate (II) Special details

**Table:**

Geometry. All esds (except the esd in the dihedral angle between two l.s. planes) are estimated using the full covariance matrix. The cell esds are taken into account individually in the estimation of esds in distances, angles and torsion angles; correlations between esds in cell parameters are only used when they are defined by crystal symmetry. An approximate (isotropic) treatment of cell esds is used for estimating esds involving l.s. planes.

### Bis(3-hyroxypyridinium) 2,5-dichloro-3,6-dioxocyclohexa-1,4-diene-1,4-diolate (II) Fractional atomic coordinates and isotropic or equivalent isotropic displacement parameters (Å^2^)

**Table:**

	*x*	*y*	*z*	*U*_iso_*/*U*_eq_	
Cl1	0.93157 (3)	0.73912 (3)	0.68834 (2)	0.01680 (8)	
O1	1.24731 (9)	0.58136 (9)	0.69329 (7)	0.01740 (16)	
O2	0.68396 (9)	0.61601 (9)	0.46095 (7)	0.01689 (16)	
O3	0.41012 (10)	0.51422 (9)	0.30475 (7)	0.01640 (16)	
H3	0.509 (2)	0.5340 (19)	0.3482 (15)	0.025*	
N1	0.48820 (11)	0.71217 (11)	0.05541 (8)	0.01529 (17)	
H1	0.566 (2)	0.7718 (19)	0.0393 (15)	0.023*	
C1	1.13017 (12)	0.54789 (11)	0.60534 (9)	0.01283 (18)	
C2	0.96428 (12)	0.60823 (12)	0.58348 (9)	0.01358 (18)	
C3	0.83396 (12)	0.56708 (12)	0.48551 (9)	0.01327 (18)	
C4	0.51167 (12)	0.66180 (12)	0.16734 (9)	0.01383 (18)	
H4	0.609229	0.691448	0.228545	0.017*	
C5	0.39288 (12)	0.56593 (11)	0.19388 (9)	0.01309 (18)	
C6	0.25163 (13)	0.52430 (13)	0.10152 (10)	0.0171 (2)	
H6	0.169400	0.457667	0.116909	0.021*	
C7	0.23181 (14)	0.58069 (13)	−0.01295 (10)	0.0192 (2)	
H7	0.135178	0.553857	−0.075971	0.023*	
C8	0.35290 (14)	0.67593 (13)	−0.03506 (9)	0.0180 (2)	
H8	0.340534	0.715121	−0.113094	0.022*	

### Bis(3-hyroxypyridinium) 2,5-dichloro-3,6-dioxocyclohexa-1,4-diene-1,4-diolate (II) Atomic displacement parameters (Å^2^)

**Table:**

	*U*^11^	*U*^22^	*U*^33^	*U*^12^	*U*^13^	*U*^23^
Cl1	0.01596 (13)	0.01889 (13)	0.01530 (13)	0.00248 (8)	0.00420 (9)	−0.00478 (8)
O1	0.0143 (3)	0.0210 (4)	0.0144 (3)	0.0017 (3)	0.0004 (3)	−0.0030 (3)
O2	0.0118 (3)	0.0224 (4)	0.0151 (3)	0.0047 (3)	0.0018 (3)	−0.0023 (3)
O3	0.0141 (3)	0.0203 (4)	0.0142 (3)	−0.0010 (3)	0.0032 (3)	0.0034 (3)
N1	0.0148 (4)	0.0160 (4)	0.0155 (4)	−0.0020 (3)	0.0050 (3)	0.0005 (3)
C1	0.0129 (4)	0.0133 (4)	0.0121 (4)	0.0010 (3)	0.0034 (3)	0.0006 (3)
C2	0.0134 (4)	0.0151 (4)	0.0122 (4)	0.0021 (3)	0.0036 (3)	−0.0021 (3)
C3	0.0132 (4)	0.0144 (4)	0.0120 (4)	0.0016 (3)	0.0034 (3)	0.0006 (3)
C4	0.0122 (4)	0.0142 (4)	0.0142 (4)	−0.0007 (3)	0.0025 (3)	0.0001 (3)
C5	0.0121 (4)	0.0127 (4)	0.0144 (4)	0.0010 (3)	0.0037 (3)	0.0001 (3)
C6	0.0129 (4)	0.0180 (5)	0.0194 (5)	−0.0033 (3)	0.0031 (4)	0.0000 (4)
C7	0.0167 (5)	0.0209 (5)	0.0169 (5)	−0.0031 (4)	−0.0001 (4)	−0.0019 (4)
C8	0.0198 (5)	0.0199 (5)	0.0132 (4)	−0.0014 (4)	0.0031 (4)	−0.0005 (4)

### Bis(3-hyroxypyridinium) 2,5-dichloro-3,6-dioxocyclohexa-1,4-diene-1,4-diolate (II) Geometric parameters (Å, º)

**Table:**

Cl1—C2	1.7409 (10)	C2—C3	1.3778 (13)
O1—C1	1.2302 (12)	C4—C5	1.3915 (13)
O2—C3	1.2735 (12)	C4—H4	0.9500
O3—C5	1.3387 (12)	C5—C6	1.3952 (14)
O3—H3	0.850 (18)	C6—C7	1.3880 (15)
N1—C4	1.3384 (13)	C6—H6	0.9500
N1—C8	1.3414 (14)	C7—C8	1.3818 (15)
N1—H1	0.891 (17)	C7—H7	0.9500
C1—C2	1.4319 (13)	C8—H8	0.9500
C1—C3^i^	1.5409 (14)		
			
C5—O3—H3	109.0 (11)	N1—C4—H4	120.1
C4—N1—C8	123.22 (9)	C5—C4—H4	120.1
C4—N1—H1	119.0 (11)	O3—C5—C4	121.95 (9)
C8—N1—H1	117.8 (11)	O3—C5—C6	119.64 (9)
O1—C1—C2	124.03 (9)	C4—C5—C6	118.41 (9)
O1—C1—C3^i^	117.27 (8)	C7—C6—C5	119.69 (9)
C2—C1—C3^i^	118.70 (8)	C7—C6—H6	120.2
C3—C2—C1	123.17 (9)	C5—C6—H6	120.2
C3—C2—Cl1	120.15 (7)	C8—C7—C6	119.88 (9)
C1—C2—Cl1	116.69 (7)	C8—C7—H7	120.1
O2—C3—C2	126.33 (9)	C6—C7—H7	120.1
O2—C3—C1^i^	115.54 (8)	N1—C8—C7	118.94 (9)
C2—C3—C1^i^	118.13 (8)	N1—C8—H8	120.5
N1—C4—C5	119.86 (9)	C7—C8—H8	120.5
			
O1—C1—C2—C3	−178.76 (10)	C8—N1—C4—C5	0.55 (16)
C3^i^—C1—C2—C3	0.78 (16)	N1—C4—C5—O3	−179.23 (9)
O1—C1—C2—Cl1	1.08 (14)	N1—C4—C5—C6	0.30 (15)
C3^i^—C1—C2—Cl1	−179.38 (7)	O3—C5—C6—C7	178.57 (10)
C1—C2—C3—O2	179.87 (10)	C4—C5—C6—C7	−0.97 (15)
Cl1—C2—C3—O2	0.03 (15)	C5—C6—C7—C8	0.83 (17)
C1—C2—C3—C1^i^	−0.78 (16)	C4—N1—C8—C7	−0.70 (16)
Cl1—C2—C3—C1^i^	179.39 (7)	C6—C7—C8—N1	−0.01 (17)

Symmetry code: (i) −*x*+2, −*y*+1, −*z*+1.

### Bis(3-hyroxypyridinium) 2,5-dichloro-3,6-dioxocyclohexa-1,4-diene-1,4-diolate (II) Hydrogen-bond geometry (Å, º)

**Table:**

*D*—H···*A*	*D*—H	H···*A*	*D*···*A*	*D*—H···*A*
O3—H3···O2	0.852 (17)	1.803 (17)	2.6277 (12)	162.5 (16)
O3—H3···O1^i^	0.852 (17)	2.438 (17)	2.9738 (12)	121.6 (14)
N1—H1···O2^ii^	0.889 (17)	1.807 (17)	2.6684 (12)	162.6 (16)
C8—H8···O1^iii^	0.95	2.45	3.1481 (13)	130

Symmetry codes: (i) −*x*+2, −*y*+1, −*z*+1; (ii) *x*, −*y*+3/2, *z*−1/2; (iii) *x*−1, *y*, *z*−1.

### Bis(4-hyroxypyridinium) 2,5-dichloro-3,6-dioxocyclohexa-1,4-diene-1,4-diolate (III) Crystal data

**Table:**

2C_5_H_6_NO^+^·C_6_Cl_2_O_4_^2^^−^	*Z* = 2
*M**_r_* = 399.19	*F*(000) = 408.00
Triclinic, *P*1	*D*_x_ = 1.671 Mg m^−^^3^
*a* = 5.49136 (13) Å	Mo *K*α radiation, λ = 0.71075 Å
*b* = 8.2195 (4) Å	Cell parameters from 11077 reflections
*c* = 18.1382 (9) Å	θ = 3.0–30.1°
α = 102.177 (3)°	µ = 0.45 mm^−^^1^
β = 93.952 (3)°	*T* = 120 K
γ = 95.316 (4)°	Platelet, brown
*V* = 793.52 (6) Å^3^	0.35 × 0.25 × 0.12 mm

### Bis(4-hyroxypyridinium) 2,5-dichloro-3,6-dioxocyclohexa-1,4-diene-1,4-diolate (III) Data collection

**Table:**

Rigaku R-AXIS RAPIDII diffractometer	4124 reflections with *I* > 2σ(*I*)
Detector resolution: 10.000 pixels mm^-1^	*R*_int_ = 0.036
ω scans	θ_max_ = 30.0°, θ_min_ = 3.0°
Absorption correction: numerical (*NUMABS*; Higashi, 1999)	*h* = −7→7
*T*_min_ = 0.890, *T*_max_ = 0.948	*k* = −11→11
12373 measured reflections	*l* = −25→25
4597 independent reflections	

### Bis(4-hyroxypyridinium) 2,5-dichloro-3,6-dioxocyclohexa-1,4-diene-1,4-diolate (III) Refinement

**Table:**

Refinement on *F*^2^	Primary atom site location: structure-invariant direct methods
Least-squares matrix: full	Secondary atom site location: difference Fourier map
*R*[*F*^2^ > 2σ(*F*^2^)] = 0.030	Hydrogen site location: mixed
*wR*(*F*^2^) = 0.080	H atoms treated by a mixture of independent and constrained refinement
*S* = 1.07	*w* = 1/[σ^2^(*F*_o_^2^) + (0.0406*P*)^2^ + 0.3136*P*] where *P* = (*F*_o_^2^ + 2*F*_c_^2^)/3
4597 reflections	(Δ/σ)_max_ = 0.001
247 parameters	Δρ_max_ = 0.75 e Å^−^^3^
0 restraints	Δρ_min_ = −0.34 e Å^−^^3^

### Bis(4-hyroxypyridinium) 2,5-dichloro-3,6-dioxocyclohexa-1,4-diene-1,4-diolate (III) Special details

**Table:**

Geometry. All esds (except the esd in the dihedral angle between two l.s. planes) are estimated using the full covariance matrix. The cell esds are taken into account individually in the estimation of esds in distances, angles and torsion angles; correlations between esds in cell parameters are only used when they are defined by crystal symmetry. An approximate (isotropic) treatment of cell esds is used for estimating esds involving l.s. planes.

### Bis(4-hyroxypyridinium) 2,5-dichloro-3,6-dioxocyclohexa-1,4-diene-1,4-diolate (III) Fractional atomic coordinates and isotropic or equivalent isotropic displacement parameters (Å^2^)

**Table:**

	*x*	*y*	*z*	*U*_iso_*/*U*_eq_	
Cl1	0.42856 (5)	0.16233 (3)	−0.07860 (2)	0.01668 (7)	
Cl2	1.13911 (5)	0.28348 (3)	0.52483 (2)	0.01730 (7)	
O1	0.03685 (16)	−0.10701 (11)	−0.14946 (5)	0.02014 (18)	
O2	0.33010 (15)	0.24516 (10)	0.08473 (5)	0.01729 (17)	
O3	1.55337 (16)	0.23416 (11)	0.62981 (5)	0.01865 (17)	
O4	1.14201 (15)	0.00027 (10)	0.38854 (5)	0.01586 (16)	
O5	0.18017 (16)	0.33437 (10)	0.25526 (5)	0.01818 (17)	
H5	0.118 (3)	0.263 (2)	0.2115 (10)	0.027*	
O6	0.74923 (17)	0.40347 (11)	0.05992 (5)	0.02044 (18)	
H6	0.617 (3)	0.350 (2)	0.0681 (11)	0.031*	
N1	0.76255 (18)	0.16991 (12)	0.35832 (6)	0.01695 (19)	
H1	0.896 (3)	0.128 (2)	0.3779 (10)	0.025*	
N2	1.04584 (18)	0.71260 (12)	0.25721 (6)	0.01696 (19)	
H2	1.114 (3)	0.777 (2)	0.2998 (10)	0.025*	
C1	0.02618 (19)	−0.05358 (13)	−0.08002 (6)	0.01355 (19)	
C2	0.19204 (19)	0.07509 (13)	−0.03554 (6)	0.01332 (19)	
C3	0.18242 (19)	0.13365 (13)	0.04220 (6)	0.01289 (19)	
C4	1.52060 (19)	0.12916 (13)	0.56903 (6)	0.01293 (19)	
C5	1.33437 (19)	0.12624 (13)	0.51055 (6)	0.01346 (19)	
C6	1.30221 (19)	0.00728 (13)	0.44252 (6)	0.01271 (19)	
C7	0.6571 (2)	0.30150 (14)	0.39505 (7)	0.0178 (2)	
H7	0.719110	0.355184	0.445342	0.021*	
C8	0.4617 (2)	0.35945 (14)	0.36116 (7)	0.0169 (2)	
H8	0.390102	0.453256	0.387366	0.020*	
C9	0.3693 (2)	0.27801 (13)	0.28719 (6)	0.0141 (2)	
C10	0.4863 (2)	0.14337 (14)	0.24949 (6)	0.0169 (2)	
H10	0.430463	0.088006	0.198898	0.020*	
C11	0.6819 (2)	0.09297 (15)	0.28659 (7)	0.0180 (2)	
H11	0.761693	0.002289	0.261273	0.022*	
C12	0.8247 (2)	0.62477 (14)	0.25631 (6)	0.0169 (2)	
H12	0.743357	0.635815	0.301370	0.020*	
C13	0.7170 (2)	0.51997 (14)	0.19096 (6)	0.0152 (2)	
H13	0.560916	0.459117	0.190415	0.018*	
C14	0.8392 (2)	0.50334 (13)	0.12492 (6)	0.0145 (2)	
C15	1.0695 (2)	0.59750 (15)	0.12741 (7)	0.0178 (2)	
H15	1.155223	0.590054	0.083294	0.021*	
C16	1.1663 (2)	0.69955 (15)	0.19462 (7)	0.0181 (2)	
H16	1.321913	0.762460	0.197088	0.022*	

### Bis(4-hyroxypyridinium) 2,5-dichloro-3,6-dioxocyclohexa-1,4-diene-1,4-diolate (III) Atomic displacement parameters (Å^2^)

**Table:**

	*U*^11^	*U*^22^	*U*^33^	*U*^12^	*U*^13^	*U*^23^
Cl1	0.01602 (12)	0.01837 (13)	0.01388 (12)	−0.00454 (9)	0.00310 (9)	0.00181 (9)
Cl2	0.01776 (13)	0.01555 (12)	0.01668 (13)	0.00527 (9)	−0.00194 (9)	−0.00109 (9)
O1	0.0204 (4)	0.0252 (4)	0.0108 (4)	−0.0062 (3)	0.0022 (3)	−0.0016 (3)
O2	0.0165 (4)	0.0179 (4)	0.0136 (4)	−0.0054 (3)	−0.0007 (3)	−0.0014 (3)
O3	0.0202 (4)	0.0181 (4)	0.0140 (4)	0.0038 (3)	−0.0031 (3)	−0.0038 (3)
O4	0.0164 (4)	0.0164 (4)	0.0129 (4)	0.0021 (3)	−0.0034 (3)	0.0005 (3)
O5	0.0183 (4)	0.0175 (4)	0.0162 (4)	0.0037 (3)	−0.0050 (3)	−0.0004 (3)
O6	0.0196 (4)	0.0230 (4)	0.0135 (4)	−0.0083 (3)	0.0008 (3)	−0.0029 (3)
N1	0.0157 (4)	0.0177 (4)	0.0181 (5)	0.0022 (3)	−0.0003 (4)	0.0057 (4)
N2	0.0177 (4)	0.0173 (4)	0.0128 (4)	−0.0016 (3)	−0.0028 (4)	−0.0006 (3)
C1	0.0126 (4)	0.0151 (5)	0.0117 (5)	−0.0008 (4)	0.0003 (4)	0.0014 (4)
C2	0.0118 (4)	0.0151 (4)	0.0121 (5)	−0.0025 (4)	0.0018 (4)	0.0023 (4)
C3	0.0120 (4)	0.0131 (4)	0.0126 (5)	−0.0004 (3)	0.0000 (4)	0.0019 (4)
C4	0.0134 (4)	0.0129 (4)	0.0116 (5)	0.0001 (4)	0.0011 (4)	0.0012 (4)
C5	0.0135 (4)	0.0128 (4)	0.0133 (5)	0.0028 (4)	−0.0001 (4)	0.0007 (4)
C6	0.0122 (4)	0.0124 (4)	0.0126 (5)	−0.0005 (3)	−0.0001 (4)	0.0020 (4)
C7	0.0200 (5)	0.0153 (5)	0.0162 (5)	0.0000 (4)	−0.0032 (4)	0.0016 (4)
C8	0.0196 (5)	0.0141 (5)	0.0154 (5)	0.0020 (4)	−0.0011 (4)	0.0003 (4)
C9	0.0144 (4)	0.0131 (4)	0.0146 (5)	0.0000 (4)	0.0006 (4)	0.0032 (4)
C10	0.0201 (5)	0.0171 (5)	0.0125 (5)	0.0027 (4)	0.0010 (4)	0.0012 (4)
C11	0.0197 (5)	0.0180 (5)	0.0170 (5)	0.0045 (4)	0.0043 (4)	0.0034 (4)
C12	0.0170 (5)	0.0198 (5)	0.0129 (5)	0.0010 (4)	0.0016 (4)	0.0015 (4)
C13	0.0132 (4)	0.0164 (5)	0.0147 (5)	−0.0011 (4)	0.0008 (4)	0.0019 (4)
C14	0.0149 (5)	0.0137 (4)	0.0129 (5)	−0.0006 (4)	−0.0006 (4)	0.0002 (4)
C15	0.0156 (5)	0.0205 (5)	0.0148 (5)	−0.0039 (4)	0.0023 (4)	0.0001 (4)
C16	0.0146 (5)	0.0191 (5)	0.0181 (5)	−0.0032 (4)	−0.0001 (4)	0.0010 (4)

### Bis(4-hyroxypyridinium) 2,5-dichloro-3,6-dioxocyclohexa-1,4-diene-1,4-diolate (III) Geometric parameters (Å, º)

**Table:**

Cl1—C2	1.7363 (11)	C4—C5	1.4165 (14)
Cl2—C5	1.7438 (11)	C4—C6^ii^	1.5426 (15)
O1—C1	1.2508 (13)	C5—C6	1.3924 (15)
O2—C3	1.2532 (12)	C7—C8	1.3722 (16)
O3—C4	1.2390 (13)	C7—H7	0.9500
O4—C6	1.2586 (13)	C8—C9	1.4035 (15)
O5—C9	1.3223 (13)	C8—H8	0.9500
O5—H5	0.907 (18)	C9—C10	1.4065 (16)
O6—C14	1.3208 (13)	C10—C11	1.3703 (16)
O6—H6	0.852 (19)	C10—H10	0.9500
N1—C11	1.3456 (15)	C11—H11	0.9500
N1—C7	1.3469 (15)	C12—C13	1.3693 (15)
N1—H1	0.918 (18)	C12—H12	0.9500
N2—C16	1.3430 (16)	C13—C14	1.4009 (16)
N2—C12	1.3511 (15)	C13—H13	0.9500
N2—H2	0.877 (18)	C14—C15	1.4126 (15)
C1—C2	1.3997 (14)	C15—C16	1.3671 (15)
C1—C3^i^	1.5410 (15)	C15—H15	0.9500
C2—C3	1.3970 (15)	C16—H16	0.9500
			
C9—O5—H5	111.3 (11)	C8—C7—H7	119.4
C14—O6—H6	107.1 (13)	C7—C8—C9	118.99 (11)
C11—N1—C7	120.85 (10)	C7—C8—H8	120.5
C11—N1—H1	114.5 (11)	C9—C8—H8	120.5
C7—N1—H1	124.6 (11)	O5—C9—C8	118.46 (10)
C16—N2—C12	121.30 (10)	O5—C9—C10	122.89 (10)
C16—N2—H2	119.3 (12)	C8—C9—C10	118.62 (10)
C12—N2—H2	119.4 (12)	C11—C10—C9	119.23 (10)
O1—C1—C2	123.43 (10)	C11—C10—H10	120.4
O1—C1—C3^i^	117.79 (9)	C9—C10—H10	120.4
C2—C1—C3^i^	118.79 (9)	N1—C11—C10	121.03 (11)
C3—C2—C1	123.56 (10)	N1—C11—H11	119.5
C3—C2—Cl1	118.10 (8)	C10—C11—H11	119.5
C1—C2—Cl1	118.31 (8)	N2—C12—C13	120.52 (11)
O2—C3—C2	125.93 (10)	N2—C12—H12	119.7
O2—C3—C1^i^	116.43 (9)	C13—C12—H12	119.7
C2—C3—C1^i^	117.64 (9)	C12—C13—C14	119.37 (10)
O3—C4—C5	124.83 (10)	C12—C13—H13	120.3
O3—C4—C6^ii^	116.58 (9)	C14—C13—H13	120.3
C5—C4—C6^ii^	118.59 (9)	O6—C14—C13	123.08 (10)
C6—C5—C4	123.72 (10)	O6—C14—C15	118.05 (10)
C6—C5—Cl2	118.75 (8)	C13—C14—C15	118.87 (10)
C4—C5—Cl2	117.52 (8)	C16—C15—C14	118.63 (11)
O4—C6—C5	126.22 (10)	C16—C15—H15	120.7
O4—C6—C4^ii^	116.09 (9)	C14—C15—H15	120.7
C5—C6—C4^ii^	117.69 (9)	N2—C16—C15	121.29 (10)
N1—C7—C8	121.23 (11)	N2—C16—H16	119.4
N1—C7—H7	119.4	C15—C16—H16	119.4
			
O1—C1—C2—C3	−179.19 (11)	C11—N1—C7—C8	1.17 (18)
C3^i^—C1—C2—C3	1.29 (18)	N1—C7—C8—C9	0.81 (18)
O1—C1—C2—Cl1	−1.30 (16)	C7—C8—C9—O5	179.64 (10)
C3^i^—C1—C2—Cl1	179.17 (7)	C7—C8—C9—C10	−2.23 (17)
C1—C2—C3—O2	178.00 (11)	O5—C9—C10—C11	179.80 (10)
Cl1—C2—C3—O2	0.11 (16)	C8—C9—C10—C11	1.75 (17)
C1—C2—C3—C1^i^	−1.27 (17)	C7—N1—C11—C10	−1.68 (17)
Cl1—C2—C3—C1^i^	−179.16 (7)	C9—C10—C11—N1	0.18 (17)
O3—C4—C5—C6	179.64 (11)	C16—N2—C12—C13	−0.23 (17)
C6^ii^—C4—C5—C6	−0.41 (17)	N2—C12—C13—C14	0.54 (17)
O3—C4—C5—Cl2	0.75 (16)	C12—C13—C14—O6	179.17 (11)
C6^ii^—C4—C5—Cl2	−179.29 (7)	C12—C13—C14—C15	−0.92 (17)
C4—C5—C6—O4	−179.11 (10)	O6—C14—C15—C16	−179.08 (11)
Cl2—C5—C6—O4	−0.23 (16)	C13—C14—C15—C16	1.01 (17)
C4—C5—C6—C4^ii^	0.40 (17)	C12—N2—C16—C15	0.33 (18)
Cl2—C5—C6—C4^ii^	179.28 (7)	C14—C15—C16—N2	−0.72 (18)

Symmetry codes: (i) −*x*, −*y*, −*z*; (ii) −*x*+3, −*y*, −*z*+1.

### Bis(4-hyroxypyridinium) 2,5-dichloro-3,6-dioxocyclohexa-1,4-diene-1,4-diolate (III) Hydrogen-bond geometry (Å, º)

**Table:**

*D*—H···*A*	*D*—H	H···*A*	*D*···*A*	*D*—H···*A*
O5—H5···O1^i^	0.905 (17)	1.640 (17)	2.5208 (13)	163.2 (17)
O6—H6···O2	0.852 (17)	1.800 (17)	2.6510 (13)	177.3 (18)
N1—H1···O4	0.919 (17)	1.810 (17)	2.7000 (13)	162.3 (16)
N2—H2···O4^iii^	0.876 (18)	2.156 (17)	2.9603 (14)	152.3 (15)
N2—H2···O3^iv^	0.876 (18)	2.176 (17)	2.8384 (14)	132.1 (14)
C7—H7···Cl2	0.95	2.81	3.4540 (12)	126
C12—H12···O3^v^	0.95	2.32	3.1541 (14)	146
C13—H13···O2	0.95	2.49	3.1685 (14)	128
C16—H16···Cl1^vi^	0.95	2.77	3.4427 (12)	128

Symmetry codes: (i) −*x*, −*y*, −*z*; (iii) *x*, *y*+1, *z*; (iv) −*x*+3, −*y*+1, −*z*+1; (v) −*x*+2, −*y*+1, −*z*+1; (vi) −*x*+2, −*y*+1, −*z*.
